# Novel penile circumcision suturing devices versus the shang ring for adult male circumcision: a prospective study

**DOI:** 10.1590/S1677-5538.IBJU.2016.0204

**Published:** 2017

**Authors:** Hu Han, Da-wei Xie, Xiao-guang Zhou, Xiao-dong Zhang

**Affiliations:** 1 Department of Urology, Beijing Chao-Yang Hospital, Capital Medical University, Beijing, China

**Keywords:** Circumcision, Male, Penis, Surgical Procedures, Operative

## Abstract

**Introduction:**

To evaluate the safety and efficacy of a novel penile circumcision suturing devices PCSD and Shang ring (SR) for circumcision in an adult population.

**Materials and Methods:**

A total of 124 outpatients were randomly assigned to receive PCSD (n=62) or SR (n=62). Patient characteristics, operative time, blood loss, return to normal activities time (RNAT), visual analogue scale (VAS), scar width, wound healing time, cosmetic result, and complications were recorded.

**Results:**

There were no significant differences in blood loss, RNAT, or complications between the two groups. There were no significant differences in the VAS scores at the operation, at 6 or 24 hours after surgery (P>0.05). The wound scar width was wider in the SR group than in the PCSD group (P<0.01). Patients in the SR group had significantly longer wound healing time compared with those in the PCSD group (P<0.01). Patients who underwent PCSD were significantly more satisfied with the cosmetic results (P<0.01).

**Conclusions:**

SR and PCSD are safe and effective minimally invasive techniques for adult male circumcision. Compared with SRs, PCSDs have the advantages of faster postoperative incision healing and a good effect on wound cosmetics.

## INTRODUCTION

The results of 3 large-scale random control tests in Africa indicate that male circumcision (MC) reduces the risks of sexually-transmitted HIV infection by 50%-60% (1-3). The accumulated evidence also demonstrates that male circumcision is capable of preventing other sexually-transmitted infections (STIs), for example, circumcision reduces the possibility of males infecting or transmitting genital ulcer disease (GUD), trichomonads and gonococcus, and decreases the risks of infection with human papillomavirus (HPV) and herpes simplex virus-2 (HSV-2) (4-6). According to Wright et al., (7), male circumcision can reduce the risk of prostate cancer by 15%. Following circumcision in young adults, participants exhibit more erection confidence (8).

Clinically, the Shang ring (SR) is widely used across the world for circumcision and is associated with the advantages of a short operating time, an obvious effect and few complications. Additionally, the SR produces good long-term cosmetic results with no significant complications or adverse effects on sexual function (9). However, the SR still has disadvantages, such as postoperative pain and reduced postoperative incision healing (10). The data regarding the clinical effects of novel penile circumcision suturing devices (PCSD) for adult male circumcision are insufficient. This study will compare the clinical effects and safety of two operation methods for male circumcision in an adult population through a randomized controlled trail.

## PATIENTS AND METHODS

### Participants and Eligibility

Our study included all the patients with redundant prepuce or phimosis requiring penile circumcision. All the patients were >18 years of age and free of penile circumcision histories. The study was approved by the ethics committee of our hospital, and every participant provided written informed consent. The patients were randomly assigned to one of two groups: PCSD or SR. The randomization was performed using computer-generated simple random Tables. The inclusion criteria for the patients were the following: (1) patients with redundant prepuce or phimosis; (2) at least 18 years old and younger than 65 years who provided informed consent; (3) willing to undergo penile circumcision; and (4) willing to be randomly assigned to the SR or PCSD operation.

**Figure 1:**
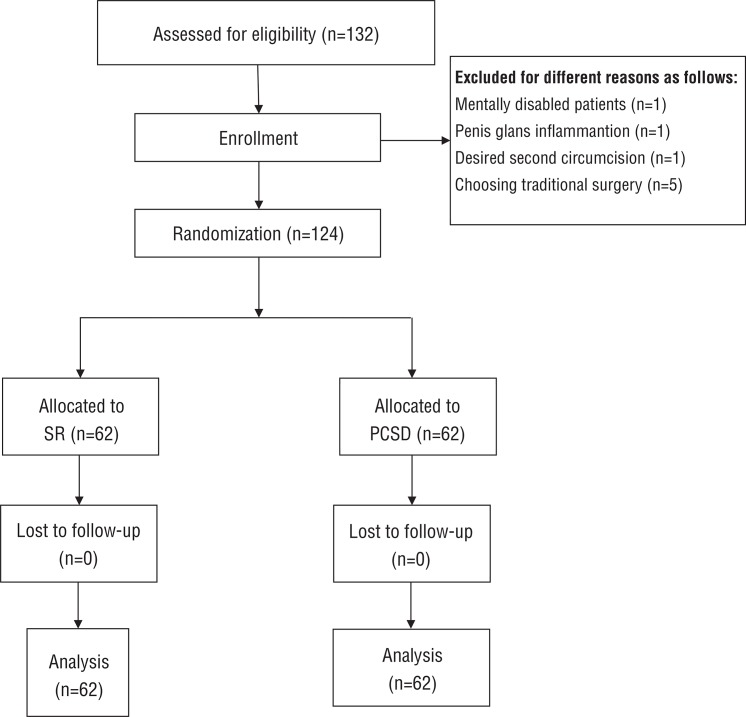
Consort diagram.

The excluding criteria were patients with the following: 1) penile malformations; 2) acute preputial balanitis; 3) HIV-positive status; 4) abnormal blood clotting function; 5) difficulty communicating, e.g., intellectual disabilities and/or low education levels; 6) unwillingness to be assigned to the SR or PCSD surgery; and 7) medically necessity for two penile circumcisions. All the selected patients conforming to all the inclusion criteria (without any of the exclusion criteria) were divided into two treatment groups and were subjected to post-operation follow-ups for at least 2 months. From February to October 2014, a total of 124 patients were invited to the study. Specific process flow charts were show in Figure-1.

### Surgical Procedures

For the patients in the SR group, a Shang ring (Wuhu Shengda Medical Treatment Appliance Technology Co.,Ltd. Wuhu City, Anhui Province, China) which is a disposable, single-use, minimally invasive device, was used. We utilized the no-flip technique that does not require the eversion of the foreskin as previously described (11). During the operation, 4 incisions were made in the incisal edge for decompression after the operation. The SR was removed with a flat plier. For the patients with phimosis, the dorsum of the penis was cut to enlarge the prepuce external orifice so that the inner ring could be easily placed between the glans and the inner preputial skin.

**Figure 2:**
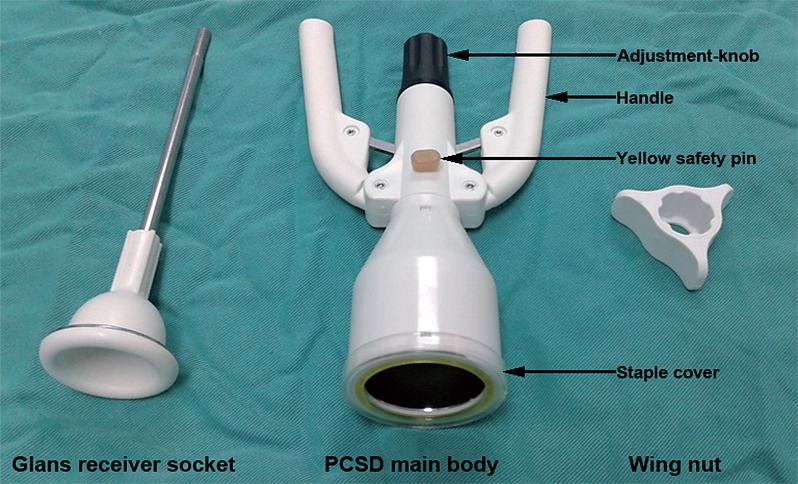
Novel penile circumcision and suturing devices.

For the patients in the PCSD group, we used the one-time PCSD invented by Changshu Henry Medical Instrument Co., Ltd. in Jiangshu China, which includes 6 models of 11-, 11+, 15, 21, 27 and 33 based on the penis circumference (Figure-2). For the selection of the model of the PCSD, the principle of “larger rather than smaller” was followed. The details of the PCSD procedure were as follows: First, the patient was placed in supine position, and the appropriate PCSD was selected according to the penis circumference. Second, disinfection with povidone iodine was applied, and the base of the penis was locally narcotized with 1% lidocaine, The PCSD of the selected size was removed from the sterile pouch, and the adjustment-knob was turned counterclockwise until the glans receiver socket could be removed. Third, the prepuce was clamped with 2-3 mosquito forceps and lifted up to place the glans receiver socket on the glans at approximately 30º of incline relative to the dorsum of penis. Fourth, the lengths of the inner and outer skin were adjusted, and the prepuce was fixed with the forefinger and middle finger of the left hand to remove the mosquito forceps. Fifth, the assistant removed the staple cover from the main body, aligned the glans receiver socket black rib with the main body rib, inserted the glans receiver socket shaft into the main body, and turned the adjustment knob clockwise via a wing nut until it stopped at the right position so that the main body was snug onto the foreskin without cutting it. Regarding stopping at the right position, the finger can be used to touch the adjustment-knob end, and if it is in the same plane, the metel shaft of glans receicer socket can be turned together with the adjustment-knob Sixth, the yellow safety pin was removed to prepare for holding the PCSD handles and squeezing evenly on both sides. The handle was then pressed to the end of its travel and held for 3-5 seconds, The handle was then released, the squeeze handle was pressed again to ensure complete cutting, and stapling was applied when necessary. Finally, the adjustment-knob was turned counterclockwise 5-8 turns to open the glans receiver socket with main body while maintaining a distance of approximately 5-6mm to determine whether foreskin adhesion was present between the glans receiver socket and main body, Pressing the foreskin with a finger cause the foreskin to fall out naturally. Because the PCSD was equivalent to a circular cutter with stapled anastomosis for circumcision (CCSAC) and a disposable circumcision suture device (DCSD), detailed descriptions of the surgical technique and figures explaining surgical procedures can be found in the studies of Yuan et al., (12), study and Lv et al., (13). For the patients with phimosis (types II, III, and IV), the dorsum was cut, and the veutro side of the penis was cut simultaneously so that the glans receiver socket could be easily placed between the glans and the inner preputial skin. The incision was sutured with 4-0 sutures to fix the foreskin on the glans receiver socket to prevent foreskin slippage and bleeding. For patients with prepuces that were not sufficiently long, absorbable sutures were used with purse-string suturing to reduce the external orifice of the prepuce so that it could be fixed on the PCSD glans receiver socket.

In both groups, preoperative, intraoperative, and postoperative parameters were determined, including age, the surgical indication for male circumcision, operative time, blood loss, the return to normal activities time (RNAT), the intraoperative and postoperative pain scores, scar width, inner plate length, time to the removal of the ring or nail, time spent removing the ring or nail, wound healing time, cosmetic results, total procedural cost, and complications were measured. The operative time was recorded from the initiation of the local anesthesia until the end of surgery. The intraoperative blood loss was calculated as follows: a completely soaked 5cm×5cm piece of gauze has an average carrying capacity of 3.25mL of blood (14).

### Follow-Up and Data Collection

All patients were advised to attend subsequent visits after surgery at 2 days, 5 days, 1 to 2 weeks and 1 month; and oral antibiotics were administered for 3 days postoperatively. The complications and wound healing times were recorded upon reexamination at 2 months postoperatively.

The pain scores were calculated via an internationally accepted visual analogue scale (VAS) pain score at the time of surgery, 6 and 24 hours after the surgery, and upon the removal of the ring or nail. We evaluated the VAS scores before oral painkillers were administered in order to reduce the deviation.

The wound scar width and the inner plate length were measured with a ruler upon the removal of the ring or nails. The inner plate length of the prepuce was defined as the length of the penile dorsal coronary groove to the incision. The wound healing time was measured from the date of surgery to the date when the wound scar was completely gone and the surgical wound had completely healed.

All patients were asked to grade the cosmetic appearance of their incision after their wound finished healing. The cosmetic results were defined on a verbal response scale. The verbal response scale had the following four options: 1, bad; 2, acceptable; 3, satisfactory; and 4, very good. The total cost of the procedure included the operative cost and medical device costs. The postoperative complications were assessed and recorded at each follow-up. Photographs were taken in preoperatively, intraoperatively, and postoperatively to document patient’s information.

## Statistical analysis

The data and results area presented as the means±the standard deviations. Statistical analysis were performed using the SPSS 18.0 statistical software package. Student’s t test, Pearson’s chi-square test and a continuity-correctioned chi-square test were used as appropriate. P values<0.05 were accepted as statistically significant. The intraoperative and postoperative data were examined with intent-to-treat analyses.

## RESULTS

### Baseline characteristics

From February 2014 to October 2014, a total of 132 outpatients were included in this study, Eight patients did not meet the inclusion criteria based on preliminary assessments, These patients included 1 case who was ruled out because he was a mentally disabled patient, 1 case was rejected for penis glans inflammation, 1 patient desired a second circumcision, and 5 patients preferred traditional surgery rather than being randomly assigned to one of the two types of surgical equipment. Ultimately, sixty-two patients were randomized to the SR approach, and 62 were randomized to the PCSD approach.

All the procedures were performed by the same urologist. Two patients experienced failed procedures due to instrument hand fracture in the PCSD group, and these failures occurred in the first ten procedures. The mean patient ages were 27.1±7.3 years in the SR group and 29.4±8.4 years in the PCSD group (P=0.115).

There was no significant difference between the two groups in terms of the indication for male circumcision (P>0.05). The patient characteristics of the two groups are illustrated in Table-1. Phimosis was classified as follows and according to Hsieh, et al., (15), study: type I (normal), the entire glans penis was visible after the retraction of the foreskin; type II (adhesion of the prepuce), the urethral meatus and part of the glans penis were visible after the retraction of the foreskin; type III (partial phimosis), the urethral meatus was visible but not the glans penis after retraction of the foreskin; type IV (phimosis), the urethral meatus and glans penis were invisisble after foreskin retraction.

**Table 1:** Baseline characteristics.

	SR	PCSD
Number	62	62
Age(year),mean ± SD	27.1±7.3	29.4±8.4
**Surgical indication(%(n))**		
Type I (normal)	87.1(54/62)	90.3(56/62)
Type II (adhesion of prepuce)	4.8(3/62)	3.2(2/62)
Type III (partial phimosis)	4.8(3/62)	4.8(3/62)
Type IV (phimosis)	3.2(2/62)	1.6(1/62)

**SR**=Shang ring; **PCSD**=penile circumcision and suturing devices

### Intraoperative and postoperative outcomes

The intraoperative and postoperative data are provided in Table-2. There were no significant differences in blood loss during the operations (0.7±0.7 vs. 1.2±1.7mL, P=0.054), RNAT (1.8±1.3 vs. 2.0±1.3 days, P=0.447) or the time to the removal of the ring or nail (10.5±1.0 vs. 10.4±1.1 days, P=0.499) between the SR and PCSD groups. The patients in the SR group had a shorter median operation time than those in the PCSD group (6.7±1.3 vs. 8.9±5.8 min, P=0.004). There were no significant differences in the VAS scores at the operation, at 6 or 24 hours after surgery, or at the removal of the ring or nail between the two groups (P>0.05).

**Table 2:** Intraoperative and postoperative outcomes.

	SR	PCSD	P value
Number	62	62	
Operative time(min),mean ± SD	6.7±1.3	8.9±5.8	0.004^a^
Blood loss(mL), mean ± SD	0.7±0.7	1.2±1.7	0.054^a^
RNAT(days),mean ± SD	1.8±1.3	2.0±1.3	0.447^a^
**VAS score, mean ± SD**			
VAS in operation	1.1±1.5	1.0±1.7	0.782^a^
VAS 6h	2.7±1.8	2.3±1.6	0.162^a^
VAS 24h	1.4±1.6	1.6±1.7	0.541^a^
VAS in removal ring or nail	5.0±2.1	5.5±2.1	0.246^a^
Scar width(mm),mean ± SD	2.8±0.4	0.9±0.5	0.000^a^
Inner plate length(cm),mean ± SD	0.9±0.9	1.4±0.5	0.001^a^
Time to removal ring or nail(days),mean ± SD	10.5±1.0	10.4±1.1	0.499^a^
Time spent removing ring or nail(min),mean ± SD	5.6±1.4	27.8±12.8	0.000^a^
Wound healing time(days),mean ± SD	30.2±4.9	15.7±3.0	0.000^a^
Cosmetic result, mean ± SD	3.1±0.6	3.7±0.5	0.000^a^
Cost(Dollars),mean ± SD	259.6±3.8	267.6±8.4	0.000^a^
Complication(%(n))			
Edema or hematoma	16.1(10/62)	8.1(5/62)	0.169^b^
Incision errhysis	0.0(0/62)	6.5(4/62)	0.127^C^
Incision dehiscence	6.5(4/62)	8.1(5/62)	1.000^C^

^**a**^ Calculated by student t test; ^**b**^ Pearson’s chi-square test was used; ^**c**^ continuity correction chi-square test was used.

**SD** = Standard deviation; **VAS** = Visual analogue scale; **RNAT** = Return to normal activities time; **SR** = Shang ring; **PCSD** = Penile circumcision and suturing devices., **1RMB** = 0.1626 Dollar.

**Figure 3:**
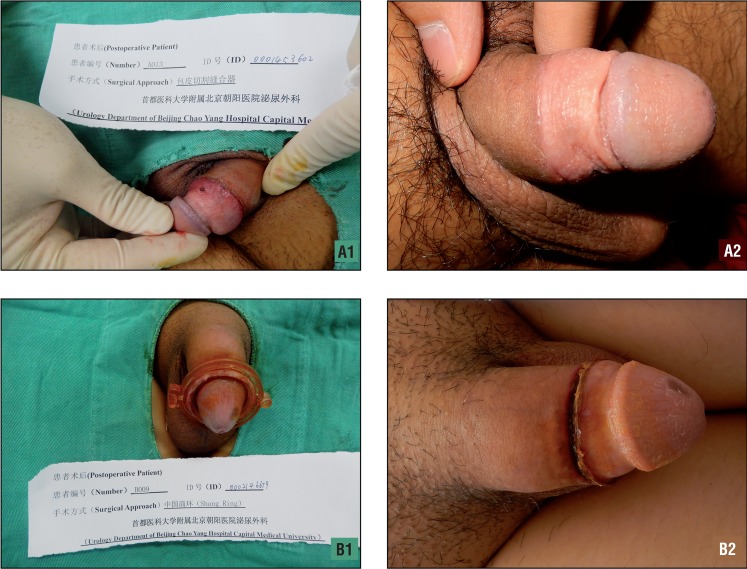
Wound healing after operation: (a1, a2) wound healing results about 3 weeks in PCSD, (b1, b2) wound healing results about 3 weeks in SR.

The wound scar width was wider in the SR group than in the PCSD group (2.8±0.4 vs. 0.9±0.5mm, P<0.01). The inner plate length of the prepuce was significantly shorter in the SR group than in the PCSD group (0.9±0.9 vs. 1.4±0.5cm, P=0.001). The patients in the SR group experienced significantly longer wound healing times that did those in the PCSD group (30.2±4.9 vs. 15.7±3.0 days, P<0.01). The times spent removing the rings or nail were significantly shorter in the SR group than in the PCSD group (5.6±1.4 vs. 27.8±12.8 min, P<0.01). The patients who underwent PCSD were significantly more satisfied with the cosmetic results as assessed with a verbal response scale (P<0.01). The cosmetic results regarding the wounds at approximately three weeks after the operations are provided in Figure-3.

The mean costs (US dollars) for the two groups were 259.6±3.8 and 267.6±8.4 (P<0.01). None of the patients in either group group experienced a wound infection. There were no significant differences in the rates of edema or hematoma, incision erhysis or incision dehiscence between the two groups. Although four incision dehiscence cases occurred at the ring removal after the operation in the SR group, none of the patients required suturing again after the operation. In contrast, 5 patients experienced partial wound dehiscence that required suturing during the operation in the PCSD group. There waswas no incision erhysis in the SR group, but 4 patients in the PCSD group experienced incision erhysis that could be alleviated by intermittent sutures or sterile gauze compression bandages during the operation. The complications among the patients in the PCSD group primarily occurred in the first 20 surgeries. Nearly every patient in the both groups exhibited involuntarily erect penises within 1 to 2 days after surgery, and this condition reduced quality of sleep at the follow-up.

## DISCUSSION

In the both groups of patients, the surgeries completed with the exceptions of 2 cases in which hand fracture occurred and necessitated changes to that were submitted open surgery in the PCSD group. The median operative time in the SR group was shorter than that in the PCSD group. There were no significant differences in blood loss, the time to return to normal activities, the time to the removal of the ring or nail, the VAS scores at the operation or 6 and 24 hours after surgery, or complications between the SR and PCSD groups. The patients in the PCSD group were more satisfied with the cosmetic aspects of the wounds than were the patients in the SR group.

The advantages of SR are high patient and provider acceptability and rates of mild adverse events that compare favorably with WHO-recommended surgical approaches (10, 16-18). The PCSD is a novel circumcision device that is based on bowel anastomotic stapler principles. The PCSD is the equivalent of a product from a different manufacturer that involves a circular cutter with stapled anastomosis for circumcision (CCSAC) reported by Yuan et al., (12), with similar operating principles.

The PCSD includes the 11-, 11+, 15, 21, 27 and 33 models. Before the operations, the PCSD models were selected based on the penis circumference and in adherence to the principle of “larger rather than smaller”. Our study indicated that the 21 and 27 models were mainly used for Chinese adults.

The median operation time in the SR group was 6.7±1.3 in our study, and this time is similar to previously described results (10, 13, 17, 18). The median operation time in the PCSD group was 8.9±5.8min, which is similar to with 7.6±4.5min reported in the study by Lv, et al., (13). In this paper, the duration of the operation equaled the sum of duration of the anesthesia of the dorsal penile nerve and the operating time. In Lv et al., (13), study, no dorsal penile nerve block was applied to the patients using disposable circumcision suture device (DCSD) and SR, but 5% lidocaine cream was applied to the surface of the penis prior to the operation. The intraoperative pain levels of the DCSD and SR group wre1.9±1.3 and 5.8±2.1, respectively, which are higher than the 1.0±1.7 and 1.1±1.5, respectively, that were observed in the present study. Therefore, we believe that the effects of superficial anesthesia are reduced compared with those of the traditional dorsal penile nerve block.

The RNAT of the patients in the PCSD group were similar to those of the patients in SR group, which is consistent with a previous study (16). Additionally, neither of the two operation methods had any effect on the regular work of the patients following the operations. Therefore, operations with either method can be completed in outpatient clinics.

Regarding the management of postoperative pain, we found no significant differences in the pain scores at 6 or 24 hours after the operations with SRs or PCSDs. The most serious postoperative pain occurred during ring and nail removal. In order to reduce the pain and enhance comfort, the patients were advised to take a painkiller or apply some topical surface anesthetic cream before coming in for ring or nail removal.

The scar width and time required for complete wound healing were significantly superior in the PCSD group. The randomized control trails of several African centers indicated that the median time to complete wound healing is 43 days in SR groups (10). Nevertheless, complete wound healing at 4 weeks was observed in 84% of patients with the ring in Rakai, Uganda (18). The scars of the patients in the SR group were wide, and the time for healing was much longer because the surface skin required healing after necrosis due to the pressing action between the inner and outer rings. Although the lengths of the inner skins of the patients in the SR group were shorter than those of the patients in the PCSD group, which may have been related to the technical level, this difference had no influence on the effects of the operations. Additionally, this factor may also have been reflected during the assessments of the two groups regarding wound cosmetics. The patients in the PCSD group felt more satisfied with the appearances of the wounds.

There was no obvious significant difference in the times to the removal of the ring or nail between the patients of the two groups. Generally, it is advised that the ring or nail be removed at approximately 10 d after the operation. Regarding ring removal at 7d, 14d or 21d after the operation, one study highlights that removal time has little effect on healing (17). However, some scholars sugested that it is better to remove the SR at approximately 2weeks after the operation so that the pain caused by ring removal can be reduced (19). In PSCD arm patients had their surgery in a mean operative time of 8.9 min. However, the procedure for removing the nail was three times longer (27.8 min). Therefore, the patients must be aware that despite better cosmetic outcomes, they will be subjected to a longer “second” surgical procedure. Luckily, based on our communications with the manufacturer about the long time required for nail removal in the patients of the PCSD group, the current PCSD has been improved so that the suturing nail can fall off automatically 3-4weeks after the operation.

For reference, the cost of dorsal slit circumcision is $17.67 and using the SR the cost is $18.21 in Zambia (20). The main disadvantage of the PCSD is that it is a one-time non-reusable device with a higher cost. In this study, there was no significant cost difference between the SR and PCSD group, in spite of a statistic difference.

During the SR procedure, the foreskin is sandwiched between the inner and outer rings before the redundant foreskin is removed. Therefore, no hematomas or exudations occurred in the majority of the SR group patients. Regarding individual patients, edema may occur due to the obstruction of lymphatic return. However, hematoma or exudation occurred in the majority of the PCSD group patients, particularly during the early stage of the application of the technique. With increased experience, we began to bandage the wound with gauze immediately after the operation and advise the patients to press the wound forcefully for approximately 5min. No wound dehiscence or bleeding occurred in any of the patients.

Wound dehiscence occurred in the SR group and PCSD groups at similar rates. Among patients in the SR group, wound dehiscence primarily occurred at ring removal after the operation, but no secondary suturing was required due to the capability for self-healing over a longer time. However, for the patients in the PCSD group, wound dehiscence primarily occurred during the operations. In this study, 5 patients exhibited partial wound dehiscences, which were discontinuously reinforced with absorbable sutures, and all the patients recovereded well after the operations. An analysis of the reason for wound dehiscence revealed that first, the adjustment-knob was not tightened during the operation, which prevented the suturing nail from completely penetrating the prepuce, and second, the wounds were was not bandaged immediately after the operations, which resulteded in hematomas and partial wound dehiscence. Among the patients in the two groups, no wound infections occurreded, which indicates that the two types of devices exhibit good bio-compatibilities with the human body.

Complications among the patients in the PCSD group primarily occurred in the early stage. Therefore, we believe these complications were strongly associated with the experience and operative skills of the surgeon. SRs and PCSDs can be used for patients with redundant prepuces and phimosis in addition to patients in whom the prepuce is not sufficiently long. During the operation, absorbable sutures can be applied with purse-string suturing to reduce the prepuce external orifice so that it can be fixed on the PCSD glans receiver socket.

The main limitations of our study are that sample size was not big enough in the PCSD group, and the follow-up was relatively short. The curative effects of PCSDs on adult patients require further clinical study for continuous confirmation. Furthermore, there is a lack of clinical studies in children patients, and this issue will be the target of our future study. In adult populations, the gold standard surgery for male circumcision is open surgery, therefore, future studies should compare PSCD with traditional surgery. Additionally, there are no comparisons on the curative effects of the PCSD devices from two Chinese companies.

## CONCLUSIONS

Generally, SR and PCSD are safe and effective minimally invasive techniques for the treatment of adult patients with redundant prepuces and phimosis. Compared with SRs, PCSDs have the advantages of faster postoperative incision healing and a good effect on wound cosmetics. Larger samples and long-term follow-up studies are needed to ascertain the clinical efficacies of PCSD devices in the future.
